# Determinants of Treatment Abandonment in Childhood Cancer: Results from a Global Survey

**DOI:** 10.1371/journal.pone.0163090

**Published:** 2016-10-13

**Authors:** Paola Friedrich, Catherine G. Lam, Geetinder Kaur, Elena Itriago, Raul C. Ribeiro, Ramandeep S. Arora

**Affiliations:** 1 Department of Pediatric Oncology, Dana-Farber/Boston Children’s Cancer and Blood Disorders Center, Boston, Massachusetts, United States of America; 2 Department of Global Pediatric Medicine, St. Jude Children’s Research Hospital, Memphis, Tennessee, United States of America; 3 Department of Oncology, St. Jude Children’s Research Hospital, Memphis, Tennessee, United States of America; 4 Institute of Child Health, University of Liverpool, Liverpool, United Kingdom; 5 Department of Medical Oncology, Max Healthcare, New Delhi, India; TNO, NETHERLANDS

## Abstract

**Background:**

Understanding and addressing treatment abandonment (TxA) is crucial for bridging the pediatric cancer survival gap between high-income (HIC) and low-and middle-income countries (LMC). In childhood cancer, TxA is defined as failure to start or complete curative cancer therapy and known to be a complex phenomenon. With rising interest on causes and consequences of TxA in LMC, this study aimed to establish the lay-of-the-land regarding determinants of TxA globally, perform and promote comparative research, and raise awareness on this subject.

**Methods:**

Physicians (medical oncologists, surgeons, and radiation therapists), nurses, social workers, and psychologists involved in care of children with cancer were approached through an online survey February-May 2012. Queries addressed social, economic, and treatment-related determinants of TxA. Free-text comments were collected. Descriptive and qualitative analyses were performed. Appraisal of overall frequency, burden, and predictors of TxA has been reported separately.

**Results:**

581 responses from 101 countries were obtained (contact rate = 26%, cooperation rate = 70%). Most respondents were physicians (86%), practicing pediatric hematology/oncology (86%) for >10 years (54%). Providers from LMC considered social/economic factors (families’ low socioeconomic status, low education, and long travel time), as most influential in increasing risk of TxA. Treatment-related considerations such as preference for complementary and alternative medicine and concerns about treatment adverse effects and toxicity, were perceived to play an important role in both LMC and HIC. Perceived prognosis seemed to mediate the role of other determinants such as diagnosis and treatment phase on TxA risk. For example, high-risk of TxA was most frequently reported when prognosis clearly worsened (i.e. lack of response to therapy, relapse), or conversely when the patient appeared improved (i.e. induction completed, mass removed), as well as before aggressive/mutilating surgery. Provider responses allowed development of an expanded conceptual model of determinants of TxA; one which illustrates established and emerging individual, family, center, and context specific factors to be considered in order to tackle this problem. Emerging factors included vulnerability, family dynamics, perceptions, center capacity, public awareness, and governmental healthcare financing, among others.

**Conclusion:**

TxA is a complex and multifactorial phenomenon. With increased recognition of the role of TxA on global pediatric cancer outcomes, factors beyond social/economic status and beliefs have emerged. Our results provide insights regarding the role of established determinants of TxA in different geographical and economic contexts, allow probing of key determinants by deliberating their mechanisms, and allow building an expanded conceptual model of established and emerging determinants TxA.

## Introduction

Treatment abandonment (TxA) is a leading cause of treatment failure for children with cancer in low- and middle-income countries (LMC).[1–5] TxA entails the failure to start or complete curative therapy (except when such treatment is contraindicated for medical reasons) and is defined by missed therapy for 4 or more consecutive weeks.[4] TxA should be distinguished from “lost to follow-up,” which is intended to describe patients who have transferred care elsewhere or have missed follow-up after completing curative therapy. Although reports on TxA in children with cancer exist since early 2000s,[6, 7] a consensus definition for TxA was not available until 2011.[4] This lack of uniformity has limited aggregated and comparative research on determinants (causes) of this complex phenomenon.

This study aimed to establish the lay-of-the-land regarding determinants of TxA globally, perform and promote comparative research, and raise awareness on this subject by capturing data directly from healthcare providers taking care of children with cancer in a variety of regional and economic settings. This study complements efforts in the global pediatric oncology community to assess the global burden of TxA,[1] evaluate published data through systematic reviews and meta-analyses,[2, 3, 5] assess the role of treatment costs on TxA in resource-limited settings,[8] and pursue on-site projects to improve TxA tracking and prevention.[9, 10] We now present our results regarding healthcare providers’ opinion on determinants of TxA and compare our results to published literature.

## Methods

### Strategy

An internet-based survey was conducted on a convenience sample in order to obtain up-to-date information from providers and centers globally. At the time this study was conducted, Cure4Kids (www.cure4kids.org) offered the broadest representation of pediatric hematology and oncology clinicians globally. Cure4Kids is a free online education and collaboration resource with diverse international membership dedicated to supporting the care of children with cancer and other catastrophic diseases worldwide.[11] Quantitative analyses of frequency, burden, and predictors of TxA were performed and reported elsewhere.[1] This report focuses on descriptive, qualitative, and landscaping analyses of healthcare providers’ opinion about causes of TxA in their setting. Queries for this study, therefore, predominantly addressed social, economic, and treatment-related factors that could influence TxA.

### Survey

An online, self-administered survey was used (see S1 Text Survey Tool to review all questions as included in the survey). The survey was evaluated for content validity by members of the International Society of Pediatric Oncology (SIOP) committee on Developing Countries (PODC) Working Group on Treatment Abandonment and piloted for ease of use in a second SIOP PODC Working Group. The survey included close- and open-ended questions, was administered in English, and required about 10–15 minutes for completion.

### Population

Physicians (including medical oncologists, surgeons, and radiation oncologists), nurses, social workers, and psychologists involved in the care of children with cancer were approached. Email addresses were obtained from the Cure4Kids member directory after ethics approval. Authors never had direct access to the master distribution list. Eligibility was confirmed through two screening questions. Students, data managers, parents and patients were excluded.

### Conducting the survey

Subjects received an individualized email-specific link, four reminders, and details regarding research activity and purpose. The survey remained open from February 10 to May 10 of 2012. Patient-level data was not collected or analyzed.

### Data Analysis

**Fig 1:**
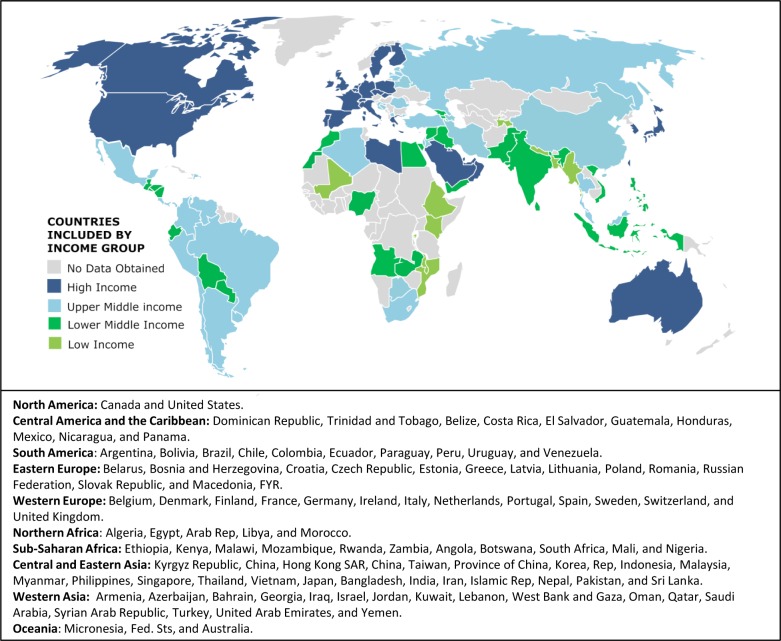
Countries included in study by World Bank income group classification in 2010 and geographical region. Country names listed are as they appear in World Bank. HIC, high-income countries; UMIC, upper-middle-income countries; LMIC, lower-middle-income countries; LIC, low-income countries. Some countries (such as Chile and Russian Federation) have a higher income group as of 2016.

Survey data was analyzed using Excel and SAS 9.3. Countries were classified according to the World Bank Atlas Method[12] by reported gross national income per capita in 2010 into high-income country (HIC), upper-middle-income country (UMIC), lower-middle-income country (LMIC), or low-income country (LIC) for the univariable and mutlivariable analyses presented in the companion manuscript.[1] These four categories were then collapsed into two categories (HIC and LMC, where LMC stands for low-and-middle income countries and integrates LIC, LMIC, and UMIC) for the descriptive, qualitative, and landscaping analyses presented in this manuscript. Of note, some countries presented in Fig 1 (such as Chile and Russian Federation) have a higher income group and some countries (such as Libya) have lower income group classification as of 2016. Because economies and their classifications change over time, for the sake of consistency, all countries were classified based on the 2010 value, regardless of values in previous or later years. Countries were also classified into 10 geographical groups (Fig 1). Demographic binary variables were analyzed with Fisher’s exact test, categorical variables with Chi-square test, ordinal variables with Spearman, and continuous variables with ANOVA or Wilcoxon Rank Test. No adjustments were made for missing data. A p-value <0.05 was considered significant. Open-ended data were independently reviewed and categorized by two investigators (P.F. and G.K.) and reviewed by a third (C.G.L.). Content analysis software was not used in the qualitative analysis. Spelling and grammar of responses were corrected only as needed for clarity; in effort to preserve respondents’ original intent, translations and any changes in wording done for clarification purposes were noted outside of quotations.

### Regulatory Requirements

This study was approved by the Institutional Review Board (IRB) at St. Jude Children’s Research Hospital and Dana-Farber Cancer Institute.

## Results

### Response Rate

The survey was sent to 3,242 email addresses. It obtained an overall contact rate of 26% and a cooperation rate of 70% for the sections of interest for this study (based on definitions established by the American Association for Public Opinion Research[13]). In particular, of 829 responses obtained (26% cooperation rate), 729 subjects met eligibility criteria, 667 provided demographic information, 581 completed domains on likelihood of TxA by diagnosis, 552 on other determinants, and 118 provided final comments (see S1 Text Survey Tool to review all sections of the survey). There were no major differences between respondents and non-respondents by country, occupation (rate of non-physicians 16% vs. 26%), and preferred language (English for 70% vs. 73%).

### Representativeness

Despite drawing from a convenience sample, the survey obtained responses from 101 countries, including all continents and country-income groups (Fig 1; 36 HIC, 29 UMIC, 26 LMIC, and 10 LIC). The 101 countries included host 85.7% of the world population 0–14 years old[1], but Africa, Oceania, and LIC were somewhat under-represented. We believe this resulted from: 1) use of internet-based English-language platform, 2) relative scarcity of providers from these contexts eligible to participate (for example, only 14 LIC and 55 LIC providers were represented in the convenience sample) and 3) low proportion of LIC economies globally (only 34 countries were classified as LIC in 2010).

### Respondents

**Fig 2:**
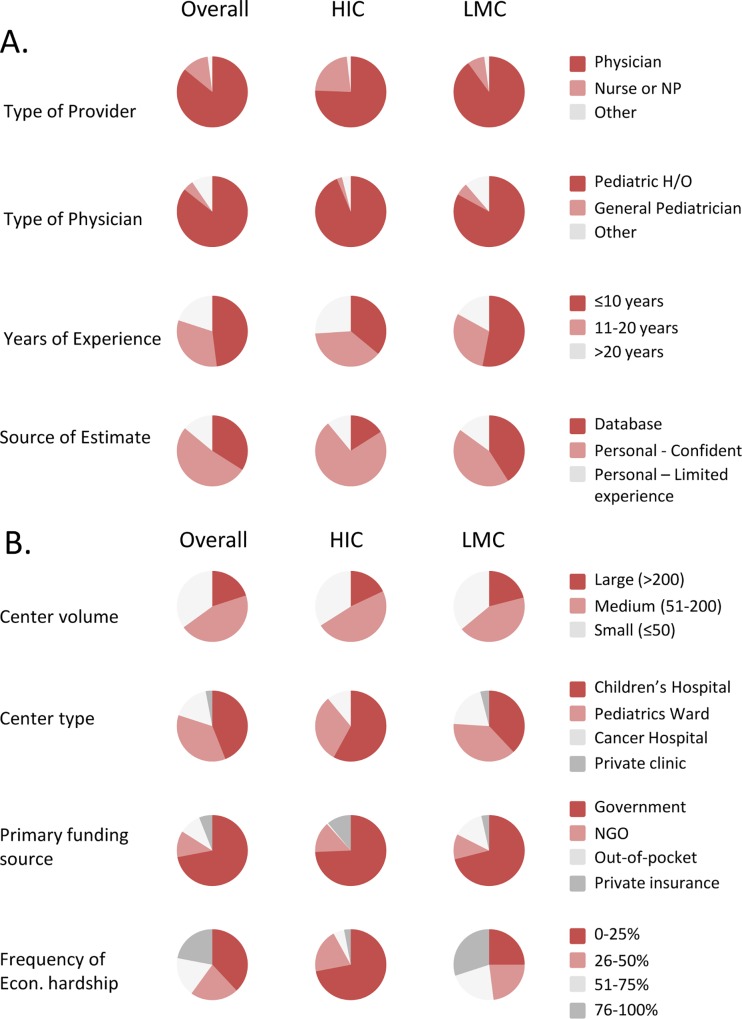
**Provider (A) and center (B) demographics.** Econ., economic; HIC, high-income countries; LMC, low-and-middle-income countries, NP, nurse practitioner; H/O, hematology/oncology; NGO, non-governmental organization. Percentages and further details of other provider and center characteristics are provided in S1 Table.

Subjects were predominantly physicians (86%); pediatric hematologists-oncologists in particular (Fig 2A; also S1 Table for frequencies and p-values). Subjects from LMC were also mostly physicians (90%; only 8% were nurses, 2% psychologists, and no social worker responded), but less exclusively pediatric hematologists-oncologists compared to HIC (83% vs. 94%, respectively). Providers from LMC more frequently reported ≤10 years of experience (53% vs. 36%) as well as greater access to a local database documenting TxA (41% vs. 16%), compared to providers from HIC. As previously reported, provider experience was the only provider characteristic independently associated with magnitude of TxA in multivariable analyses; younger providers reported higher rates of TxA.[1]

### The Centers

Most respondents (65%) worked in medium to large centers and only 3% of subjects reported working in private clinics (Fig 2B; see S1 Table for details on frequencies and p-values). Distribution by center volume was similar between LMC and HIC, but Children’s Hospitals were less common in LMC than HIC (38% vs. 58%, respectively). While government funding was the main source of funding overall (72%), reliance on out-of-pocket expenses as the primary source of funding was higher in LMC than HIC (14% vs. 0.6%, respectively). The proportion of families experiencing economic hardship at the center (defined as living below the poverty line or having significant financial challenges) was high in LMC (75%), but also relatively high in HIC (28%). As previously reported, among center characteristics assessed, the country’s income category, the center’s reliance on out-of-pocket payments as primary source of funding for treatment and, to a lesser extent, higher prevalence of economic hardship, were identified as independent predictors of TxA ≥6% in multivariable analyses.[1]

### Determinants of TxA

#### Diagnosis

**Fig 3:**
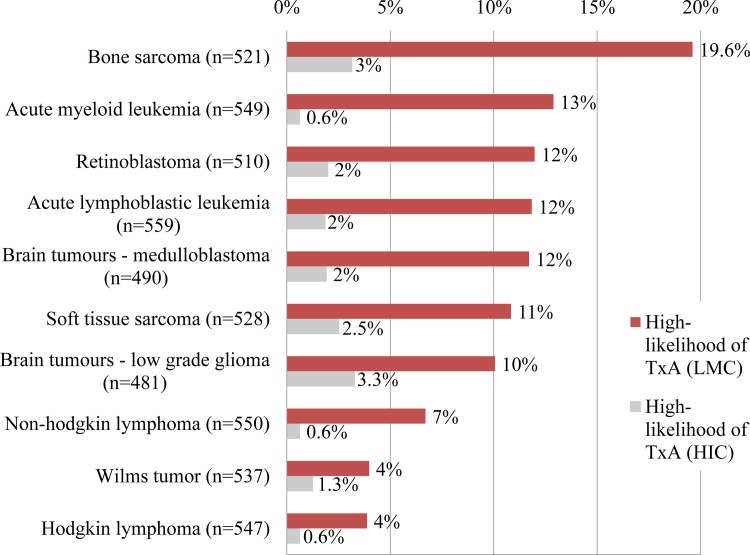
Likelihood of TxA by diagnosis. High-likelihood of TxA entailed report of TxA occurring “often” or “always/almost always”. Variable sample size results from provision of a “Don’t know” option in which case the subject was removed from the denominator. HIC, high-income countries; LMC, low- and middle-income countries; TxA, treatment abandonment; n, refers to the number of responses for each diagnosis.

We explored the role of diagnosis as a determinant of TxA. Subject were asked to report on the *likelihood* of TxA at their center for 10 individual diagnostic groups using an ascending scale: “never/almost never”, “rarely”, “sometimes”, “often”, and “always/almost always”. For each of the 10 diagnoses analyzed, the overall *likelihood* of TxA increased as the country’s World Bank income category decreased (p<0.0001 in each case). The categories “always/almost always” and “often” were aggregated to reflect *high-likelihood* of TxA. A “not applicable” option was available and it was removed from the denominator, resulting in a variable effective response rate by diagnosis; while retaining comparability across diagnoses. As seen in Fig 3, the likelihood of TxA varied by diagnosis. In LMC, *high-likelihood* was reported most frequently for bone sarcomas (20% of providers) and least frequently for Hodgkin disease and Wilms tumor (4%), although several diagnoses shared a similar range (10–13%); acute myeloid leukemia, retinoblastoma, acute lymphoblastic leukemia, brain tumors, and soft tissue sarcomas. Each diagnosis frequency’s ranking and range varied depending on the country’s income category (S1 Fig). Differences in ranking by income category were most notable for retinoblastoma, medulloblastoma, and acute myeloid leukemias between the LMIC and UMIC subgroups; UMIC ranked retinoblastoma high for *high-likelihood* of TxA, while LMIC ranked it low. Interestingly, medulloblastoma and acute myeloid leukemias showed a reverse trend (S1 Fig). Finally, as expected, the range of reported *high-likelihood* of TxA by diagnosis increased as the country group income decreased; 1–3% in HIC and 1–10% UMIC to 7–31% in LMIC and 0–50% in LIC (S1 Fig). These findings suggest the role of diagnosis, as a determinant of TxA is sensitive to the overall socioeconomic context.

### Treatment phase

**Fig 4:**
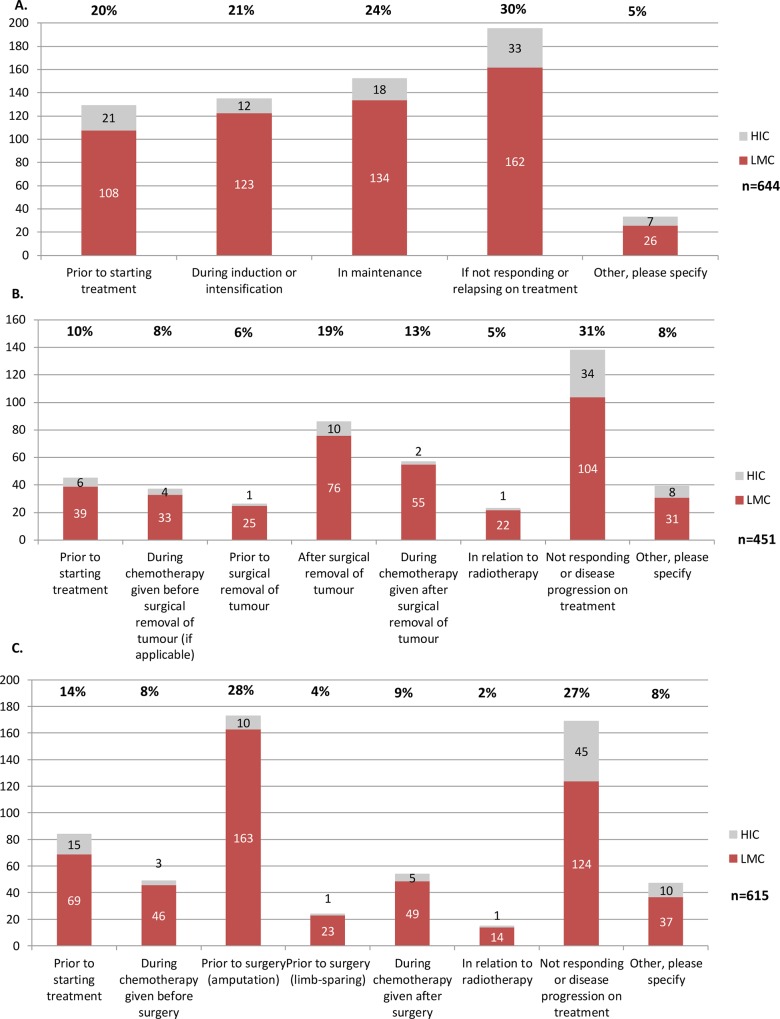
Risk of TxA by treatment phase for three diagnostic scenarios. **A: Acute lymphoblastic leukemia. B: Wilms tumor. C: Bone sarcoma.** Each provider could mark up to 3 responses. Variable response rate results from provision of “not applicable” category, in which case response was removed from denominator. HIC, high-income countries; LMC, low- and middle-income countries; n, refers to the number of responses. Percentages at the top reflect distribution across columns. Values within the columns reflect count distribution by country-income group; HIC vs. LMC.

In order to assess mechanisms of TxA, providers were presented with scenarios for acute lymphoblastic leukemia, Wilms tumor, and bone sarcoma and asked to select up to three stages in treatment during which risk of TxA was highest. Scenarios were selected based on a face validity exercise (see methods). Providers could respond “not applicable” and these responses were removed from the denominator. Across all three scenarios, high-risk of TxA was most frequently reported for children not responding to treatment or experiencing disease progression (27–31% of responses, Fig 4), particularly in HIC. In acute lymphoblastic leukemia, high-risk of TxA was otherwise similar between pre-treatment, induction, and maintenance therapy phases (20–24%; Fig 4A). However, by income group, TxA during acute lymphoblastic leukemia induction or intensification was reported with higher frequency by LMC providers (22%) than HIC providers (13%). In the case of Wilms tumor, the period after surgical removal of the tumor was also considered high-risk, particularly in LMC (19% of overall, 15% in HIC and 20% in LMC; Fig 4B). Very few providers from HIC reported TxA to occur in Wilms tumor before or after surgical resection (1.5% and 3%, respectively), compared to providers from LMC (6.5% and 15%, respectively). Finally, for bone sarcomas, the pre-amputation period was considered high-risk, particularly in LMC (28% overall, 11% in HIC and 31% in LMC; Fig 4C). Free-text responses manually reviewed supported the distributions described. Therefore, either the treatment phase itself or perceived prognosis appeared to influence the identified high-risk periods.

### Socioeconomics, beliefs, preferences, comorbidities, and others

Subjects were asked how each of 15 factors would influence the *likelihood* of TxA in their setting. The S2 Fig shows the factors assessed and their distribution by income category. The categories “increases” and “strongly increases” were aggregated to describe factors perceived to increase the *likelihood* of TxA. All factors except older age and male gender were perceived as significantly more influential in LMC as in HIC (only older age and male gender had p-value >0.05, see S2 Fig). Based on the frequency and ranking of each factor, five patterns were identified.

1. Factors perceived to play a major role in LMC, but comparatively lower in HIC: low socioeconomic status, low parental education, and long travel time to center (these three are here on referred to as social/economic factors).
2. Factors perceived to play an important role in both LMC and HIC: preference for complementary and alternative medicine (CAM) and concern for adverse effects and toxicity.
3. Factors perceived to play a moderate role in HIC, but comparatively lower in LMC: strongly held faith or religious beliefs and older age/adolescent.
4. Factors perceived to play a moderate role in both LMC and HIC: belief in incurability of cancer, insufficient communication, and painful diagnostic or therapeutic procedures.
5. Factors perceived to play minor role, but comparatively higher in LMC than HIC: malnourishment, HIV status, younger age, and female gender.

**Fig 5:**
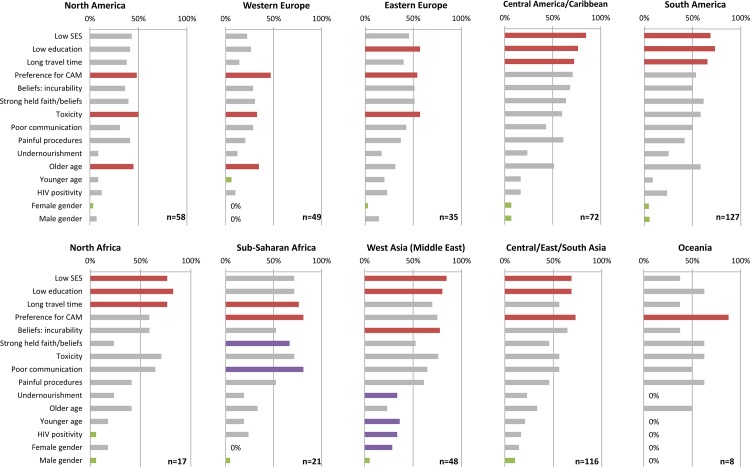
Report of high-likelihood of TxA by specific factors and geographic group. Positive responses arise from affirmative response to “increases” or “strongly increases” likelihood of TxA. The factors with the 3 highest frequencies for each region are reported in red. Factors with lowest frequency in green, except if response = 0%. Purple highlights interesting response pattern in the Middle East and Sub-Saharan Africa as discussed in the text. SES, socioeconomic status; CAM, complementary and alternative medicine.

The same 15 factors were analyzed by geographical group (Fig 5) looking for regional differences in their appraisal. Responses from North America and Europe showed preference for CAM, concerns about toxicity, and older age as important factors in these regions. Parental education was perceived of higher influence in Eastern than Western Europe. Responses from Central and South America gave most importance to social/economic factors, followed by beliefs, toxicity, older age, and preference for CAM. Responses from North and Sub-Saharan Africa contrasted somewhat in their response patterns; while both groups weighted socioeconomic factors and toxicity highly, those from Sub-Saharan Africa gave added weight to preferences, beliefs, and communication. Responses from West Asia (Middle East) rated most factors relatively high for their country income level. Of particular interest was the high rating of factors related to therapy (beliefs about incurability and concerns about toxicity) and factors related to vulnerable populations (younger age, HIV positivity, and female gender). Central-East-South Asia followed a similar pattern as Central-South America. Oceania (the smallest region analyzed) ranked preference for CAM as the dominant factor increasing TxA. Therefore, although social/economic factors achieved the highest ranking among LMC in the analysis by country income group, regional patterns were also readily identified and likely reflect cultural differences between providers and/or regions.

**Fig 6:**
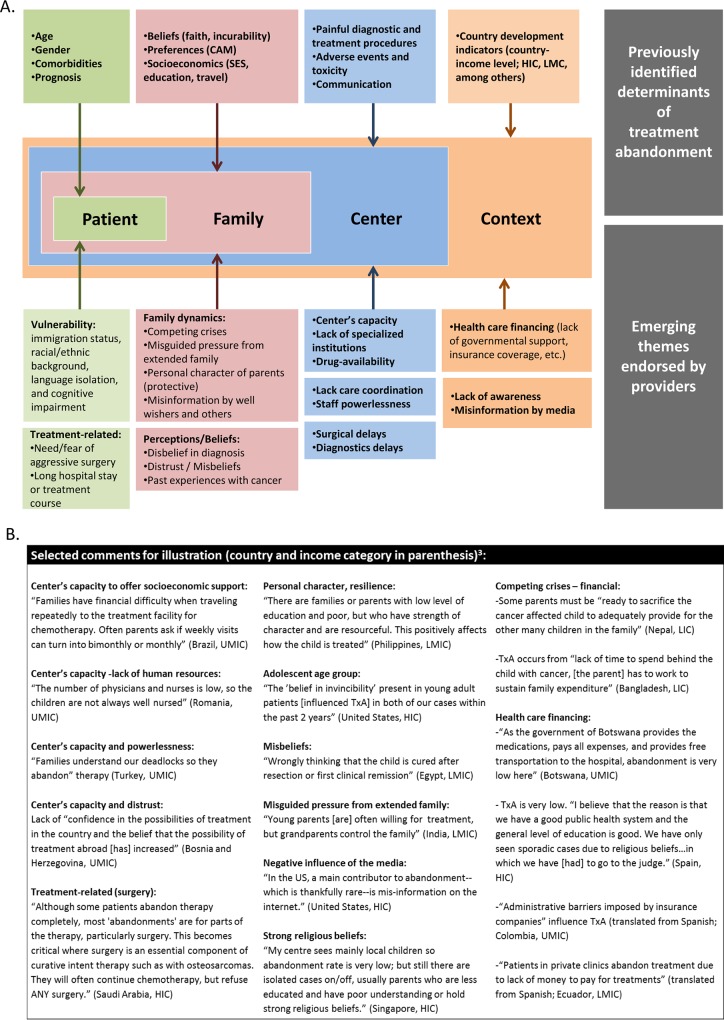
**New and previously identified factors of TxA (A) and supporting statements (B).** CAM, complementary and alternative medicine. HIC, high-income countries; UMIC, upper-middle-income countries; LMC, low- and middle-income countries; LMIC, lower-middle-income countries; LIC, low-income countries. Details regarding frequency of each factor are available in S2 Table.

In an effort to identify additional and/or emerging factors, subjects were asked to provide comments and to suggest factors that influenced the *likelihood* of TxA in their setting. The summary from 194 interpretable responses provided by 104 subjects is presented in Fig 6 along with selected illustrative comments. All previously established or reported factors are listed at the top of the ecologic model and all newly identified or emerging factors below the ecologic model. A more detailed description of each construct and its frequency is also available (S2 Table). Most of the factors addressed in close-ended queries were supported by open-ended queries and free-text comments. Recurrent themes included: a) contextual factors such as the issue of healthcare financing, b) center and care delivery-related factors such as the negative impact of poor infrastructure and limited human resources at the center, c) family factors such as competing family crises and problematic family dynamics, and d) patient factors related to vulnerability or treatment, such as immigration status and need for/fear of aggressive surgery, respectively. Themes not previously reported included the protective impact of personal character and the negative impact of belonging to vulnerable populations, such as discriminated, native/indigenous, or immigrant populations. Immigration status was identified as a factor specifically important in UMIC and HIC, where other factors are presumably lessened.

Although comments from providers from HIC were less frequent, they expressed particular concerns regarding contextual factors such as lack of healthcare coverage for immigrant populations and the negative impact of mis-information in the media; care delivery factors such as informed consent for therapy when language barriers exist, and the issue of respecting adolescent autonomy in medical decision-making (particularly when there is refusal of certain aspects of treatment); and family and patient-related factors, such as parental worries regarding unexpected side effects, the impact of strong religious beliefs or preference for CAM, and the issue of non-adherence with oral medications.

## Discussion

Treatment abandonment (TxA) is complex and multifactorial, but understanding and addressing it is vital to bridge the survival gap between HIC and LMC. This is the first study to collect data directly from healthcare providers taking care of children with cancer in a wide variety of regional and economic settings globally. Our results provide valuable insights regarding the role of recognized determinants of TxA such as diagnosis, treatment phase, prognosis, social/economic factors, and beliefs in different geographical and economic contexts. Results also allow probing key established determinants by deliberating their mechanisms and building an expanded conceptual model that takes into account established and emerging patient, family, center, and context factors that influence the risk of TxA (Fig 6).

### Diagnosis

Our results show variability in the likelihood of TxA by diagnosis (high for bone sarcomas and low for Hodgkin lymphoma and Wilms tumor), with the range of this TxA likelihood and the ranking of specific diagnoses varying by socioeconomic context; a finding consistent with other studies documenting higher rate of TxA for specific diagnoses (sarcomas, retinoblastoma, etc.).[2, 14–16] However, experience demonstrates that when baseline income inequalities and frequency of TxA are high, a significant number of patients with lymphoma and Wilms tumor abandon therapy.[17–24] For these common and curable childhood cancers, even small percentages of TxA may be significant in crude numbers. Furthermore, there is no inherent mechanism by which having a specific diagnosis should cause TxA. The association is most likely mediated by determinants such as the social/economic context, beliefs, strategies needed or available for cure, and prognosis. Therefore, documentation of differences in risk or burden of TxA by diagnosis at centers should be interpreted taking into account the overall frequency of the disease as well as the overall frequency of TxA.

We believe documenting this differential in the likelihood of TxA by diagnosis offers two take-home messages. First, centers should track TxA by disease (and not assume that the frequency of TxA observed for one disease applies to other diseases) in order to identify patient populations at higher risk of TxA or for whom the current strategies to prevent TxA are not working. Second, as interventions and priorities are established, centers should keep in mind the potential untapped opportunities to increase survival outcomes through TxA prevention in children with curable cancer.

As previously mentioned, we hypothesized that social/economic context, aggressiveness of the treatment strategy, and prognosis could serve as mediators between diagnosis and TxA. Providers’ comments supported our hypothesis. For example, the frequency rankings varied by country income group, the need for aggressive and/or mutilating surgery was repeatedly suggested to play a direct role, and the highlighting of specific diagnoses often reflected a comment on prognosis (for example “infant with CNS tumor” or “metastatic sarcoma”). The analysis also revealed the center’s capacity (including human resources, supportive care, and drug-availability, among others) and perceived (rather than actual) prognosis as additional mediators. A recent assessment of pediatric oncology centers in Myanmar showed higher TxA for retinoblastoma compared to other oncologic diseases and supported the idea that lack of specialists, radiation services, and supportive care contribute to TxA for this diagnosis in their setting.[14] Perceived poor prognosis, by parents or providers, as a result of failed communication and education strategies are emerging determinants of TxA.[25, 26]

### Treatment phase

The role of treatment phase as a determinant of TxA was considered of interest because most studies show TxA to occur early–in the first 3 months of therapy.[16, 27–31] TxA occurring predominantly at later stages of treatment has only been reported in the context of hospital detention policies that impede a patient from leaving the hospital until the bill has been paid.[32–34] However, higher risk of TxA at other phases of treatment (including maintenance therapy in leukemia, following removal of Wilms tumor, and prior to amputation in sarcomas) has also been reported.[16, 23, 31, 35] In our study, risk of TxA was reported as highest either when the prognosis clearly worsened (lack of response to therapy, relapse), when the general appearance of the child could allow parents to perceive the prognosis as favorable (induction completed, mass removed), or when aggressive/mutilating surgery was proposed. Therefore, as seen for diagnosis, the mechanism for treatment phase as a determinant of TxA appeared more closely related to perceived prognosis than the treatment phase per se. Although challenging in busy, over-crowded clinics and wards, developing strategies for appropriate communication of treatment plans, expectations, and events may be a cornerstone for reducing TxA.

### Established determinants

Based on published literature, several other determinants of TxA were assessed (toxicity, beliefs, pain, etc.). Providers from LMC placed social/economic factors at the top. This finding is consistent with single-institution retrospective studies showing economic constraints (low income or financial difficulties), low parental education (or literacy), and long travel time to be associated with increased risk of TxA[25, 27, 28, 30, 36–42] and treatment-related mortality.[43–45] Interestingly, by region, Sub-Saharan Africa, where 26 of the 34 poorest countries in the world are located, ranked poor communication and preference for CAM, rather than socioeconomics, at the top. Studies from Kenya support these findings, prioritizing poor communication as a determinant of TxA.[25, 33]

Providers from HIC and LMC concurred on preference for CAM and concerns regarding treatment toxicity as important; in aggregate, these ranked highest in HIC and second highest in LMC. A possible increase in refusal arising from the appeal of CAM in HIC has been postulated,[46] supported by a survey of clinics in Germany documenting annual incidence of TxA at 0.5% and reporting parents’ beliefs as the main reason for refusal or discontinuation of treatment.[47] Therefore, in HIC, TxA as a result of preference for CAM has often been related to families’ efforts to reduce toxicity.[48] CAM is broadly used in LMC,[49] but provider appraisal of its role as a determinant of TxA in LMC had not been thoroughly evaluated. Interestingly, interviews with parents suggest preference for CAM in LMC may more closely relate to supporting community beliefs, managing symptoms, and searching for more affordable or accessible alternatives, and not necessarily to a focus on reducing toxicity.[50] In LMC, intensity of treatment appears to be a double-edged sword with side effects perceived by some parents as proof of efficacy,[50, 51] but a major source of concern for others.[51, 52]

Other factors ranked as contributing to TxA included belief in the incurability of cancer, insufficient communication, strongly held faith or religious beliefs, and painful procedures; determinants supported by several single institution studies.[27, 30, 38, 40, 42] The importance attributed to age, gender, nutrition, and HIV status was overall lower, but of greater importance in LMC than HIC. In this study, it was predominantly providers from West Asia (Middle East) who demonstrated a particular concern for vulnerable populations (based on nutritional status, age, gender, chronic illness, or immigration status) as a determinant of TxA. Of these, only malnutrition has been clearly reported to influence outcomes in LMC through correlation with prolonged neutropenia[53] and deaths due to TxA and treatment failure.[54] The impact of dose-modification, supervised nutritional supplementation, increased awareness and high vigilance for this patient population remains to be determined but is likely to be beneficial. The role of gender has been infrequently documented[30] and despite the historical stigma of HIV, particularly in Sub-Saharan Africa, higher TxA as a result of concurrent HIV infection has not been reported for children with cancer.

### New themes and conceptual model

With increased recognition of the role of TxA on global pediatric cancer outcomes,[55] factors beyond social/economic factors and beliefs have emerged. Using free-text comments from providers, we were able to assess and expand our conceptual model of TxA to include a broader range of emerging individual, family, center, and contextual factors (Fig 6).

1. Vulnerability–Immigration status was brought up by providers from HIC, who described poor access to care when these children do not qualify for national health care coverage. Long-hospital stay and treatment course were also endorsed (presumably as a result of the additional time and financial burden they impose).
2. Family dynamics–The role of family dynamics and the connection between families and their communities were of particular interest. Studies show parents are motivated to cure their child with cancer, even in very low resource settings.[24, 50] However, in a recent study from Kenya, interviews with parents who abandoned treatment showed a large proportion of parents to be ill-advised by their community (74% of parents had been advised to seek alternative treatment and 54% to stop medical treatment).[32] Without the balancing act of good communication strategies by providers and social/economic supports to complete therapy (through governmental or non-governmental program assistance), it should be no surprise if families opt to follow the guidance provided by their established social networks.
3. Perceptions–Public perception of cancer is likely very different on HIC and LMC. In HIC, investment by private citizens in fundraising and awareness campaigns for cancer (and childhood cancer in particular) has been strong for decades, allowing cancer to inspire individual resilience and social thriving. In LMC, where the burden of cancer mortality is high and public awareness campaigns are relatively young, a diagnosis of childhood cancer may be poorly accepted or understood. The role of beliefs as a determinant of TxA presented by providers went beyond religiosity or disbelief in curability of cancer. Providers highlighted disbelief in the center’s capacity and past family experiences with cancer as additional factors influencing the risk of TxA.
4. Center’s capacity: This was highlighted as a determinant of TxA in terms of human resources, infrastructure, supportive care, and internal health delivery systems. Most studies looking at determinants of TxA focus on the family. However, the role providers and centers play in swaying this phenomenon are emerging[24, 26, 40, 52] and the benefits of an integral and multidisciplinary approach have been documented.[56–58] A shift in focus from static determinants of TxA (age, gender, diagnosis, prognosis, etc.) to more actionable factors such as perceived prognosis, communication, center’s capacities, and public awareness, allows shifting from traits we can’t necessarily control, to areas we can improve.
5. Context: The issue of healthcare financing for catastrophic illnesses and the need to protect families from financial suicide is one that burdens policy makers in HIC and LMC.[59] Regarding TxA, lack of governmental support has been associated with higher rate of TxA.[34, 60–62] Incomplete coverage by private insurers and administrative barriers imposed by insurance companies were additional factors raised by providers. Finally, as discussed in the context of preference for CAM and beliefs, lack of awareness and misinformation by the media were postulated by providers to play a role in LMC and HIC, respectively. Continuing to explore how the overall social context directly or indirectly influences TxA through policies, awareness, and perception remains of interest.

In conclusion, TxA is a complex and multifactorial phenomenon. Our results provide valuable insights regarding the role of recognized determinants of TxA in different geographical and economic contexts. Results also allow probing of key determinants by deliberating their mechanisms and building an expanded conceptual model that takes into account patient, family, center, and context factors that influence the risk of TxA.

Regarding the limitations of our study, by using an online English-language platform and drawing from a convenience sample, we likely lowered the chances of receiving information from LIC and possibly selected for more motivated individuals. However, when this study was conducted, the Cure4Kids online membership offered the largest and most diverse cohort of pediatric hematology and oncology providers available to conduct this study. Although not fully representative, the sample achieved was sufficient to meet the exploratory aims of the study. Furthermore, contact and cooperation rates achieved were comparable to other global surveys.[63–66] We also acknowledge the limitations inherent to the survey research methodology including the need to rely on standardization, possible recall bias, and the lack of a confirmatory source in particular. Mindful of these methodological limitations, doing this study has allowed us to explore in great detail, determinants of treatment abandonment which are currently relevant at a global level and explores regional variations in these determinants. We hope our results promote further comparative research on the subject of TxA and its determinants globally.

## Supporting Information

S1 Table Self-reported subject and center characteristics (PDF)

S2 Table Determinants of TxA as reported by providers on free-text comments (PDF)

S1 Fig Report of high-likelihood of TxA by diagnosis and country income group. Dark blue, HIC = high-income countries; light blue, UMIC = upper-middle-income countries; light green, LMIC = lower-middle-income countries; dark green, LIC = low-income countries; HL = Hodgkin Lymphoma, NHL = Non-Hodgkin Lymphoma; WT = Wilms tumor; RB = Retinoblastoma; STS = Soft tissue sarcoma; ALL = Acute lymphoblastic leukemia; GLIOMA = Brain glioma; AML = Acute myeloid leukemia; BS = Bone sarcoma; and MB = Medulloblastoma.
(PDF)

S2 Fig Likelihood of TxA by specific factors and country income group. The category “increases likelihood of TxA” entailed report of “increases” or “strongly increases” likelihood of TxA. HIC, high-income countries; LMC, low- and middle-income countries; TxA, treatment abandonment; CAM, complementary and alternative medicine.
(PDF)

S1 Text Survey Tool. The data presented in this manuscript pertains primarily to questions 1–12, 14–20, and 32 of the survey tool used for data collection. Results for other sections have already been (see text) or will be summarized in additional manuscripts.
(PDF)
