# Supplementary material for: Determinants of Treatment Abandonment in Childhood Cancer: Results from a Global Survey
Source: PLoS One. 2016 Oct 13;11(10):e0163090. doi: 10.1371/journal.pone.0163090 (PMC5063311; doi:10.1371/journal.pone.0163090)
Supplement: S1 Table — (PDF) [file pone.0163090.s001.pdf]

S1 Table. Self-reported subject and center demographics

	Total		HIC		LMC		
**Responses**	**581**		**168**		**413**		
**Countries**	**101**		**36**		**65**		
	**n**	**%**	**n**	**%**	**n**	**%**	**p-value**
**Occupation**							
Physician	499	85.9	127	75.6	372	90	
Nurse/nurse practitioner	70	12	38	22.6	32	7.8	
Social Worker	1	0.2	1	0.6	0	0	**<0.0001**
Psychologist	8	1.4	0	0	8	2	
Other**	3	0.5	2	1.2	1	0.2	
**Type of Physician**							
Pediatric H/O	423	85.7	118	93.6	305	83	
Adult H/O	14	2.8	1	0.8	13	3.5	
General Pediatrician	24	4.8	3	2.4	21	5.7	0.057
General Physician	2	0.4	0	0	2	0.5	
Other[#]	31	6.3	4	3.2	27	7.3	
**Experience**							
5 years and less	140	24	26	15	114	28	
6 to 10 years	140	24	35	21	105	25	
11 to 15 years	96	17	36	21	60	15	**0.003**
16 to 20 years	89	15	28	17	61	15	
More than 20 years	116	20	43	26	73	17	
**Sex**							
Female	327	56	91	54	236	57	0.5
**Provided estimate of TxA is from**							
Local database	194	34	26	16	168	41	
Personal opinon, but confident	304	52	123	73	181	44	**<0.0001**
Personal opinion, but not experienced	83	14	19	11	64	15	
**Center volume (new cases/year)**							
25 and less	87	15	27	16	60	14	
26 to 50	120	20	30	18	90	22	
51 to 100	127	22	39	23	88	21	0.7
101 to 200	132	23	42	25	90	22	
More than 200	115	20	30	18	85	21	
**Center type**							
Children's hospital	253	44	97	58	156	38	
Cancer hospital	101	17	18	11	83	20	**<0.0001**
General hospital (children's ward)	207	36	52	31	155	38	
Private clinic	17	3	0	0	17	4	
**Primary funding source**							
Government	411	72	123	74.4	288	71	
Private insurance	33	6	18	11	15	3.5	**<0.0001**
Out-of-pocket by family	58	10	1	0.6	57	14	
Non-governmental organization	70	12	23	14	47	11.5	
**Secondary funding source**							
Government	140	26	43	29	97	25	
Private insurance	109	20	54	36	55	14	**<0.0001**
Out-of-pocket by family	82	15	15	12	64	17	
Non-governmental organization	206	39	34	23	172	44	
**Economic hardship at center****							
0-25%	203	38	106	72	97	25	
26-50%	118	22	29	20	89	23	**<0.0001**
51-75%	92	18	7	5	85	22	
>75%	118	22	5	3	113	30	

[#]Other includes: surgeons (17), radiation therapists (2), palliative care specialists (3), intensivist (1), infectious disease specialists (6), epidemiologist (1), and anesthesiologist (1).

**Economic hardship was asked as a free-text question with range 0-100% regarding the proportion of indigent families (below the poverty line) at the center.
