# Supplementary material for: Determinants of Treatment Abandonment in Childhood Cancer: Results from a Global Survey
Source: PLoS One. 2016 Oct 13;11(10):e0163090. doi: 10.1371/journal.pone.0163090 (PMC5063311; doi:10.1371/journal.pone.0163090)
Supplement: S2 Table — (PDF) [file pone.0163090.s002.pdf]

S2 Table. Determinants of TxA as reported by providers on free-text comments[1]

**Additional factors suggested by providers in the free-text comments[2]:**		
THE CENTER<br>-**center's capacity** (15) – lack of treatment choices, human resources, housing, transportation, social worker, beds, blood products, adequate supportive care measures<br>-**drug-availability** (3)<br>-**physician powerlessness** (3) – regarding parental awareness of deadlocks at center, futility of care, center's capacity<br>-**surgical delays** (3) – delays in proceeding to pediatric, neurologic and orthopedic surgery<br>-**diagnostic delays** (2)<br>-**lack of coordinated care** (2) – lack of coordinated approach to treatment and coordination of care between radiation oncology and medical oncology<br>-**lack of specialized institutions** (1)<br><br>TREATMENT<br>-**prognosis** (6) – poor prognosis, metastatic disease, severity of illness, infant with CNS tumor, sense of incurability<br>-**need/fear for aggressive surgery** (6) – fear of amputation, enucleation, disfiguring surgery, or mutilating surgery<br>-**progression / relapse** (5) – refractoriness, progression, multiple relapses<br>-**diagnosis** (1) – more common in solid tumors compared to hematological malignancies<br>-**long hospital admissions** (1)<br>-**long treatment course** (1)	SOCIAL/FAMILY DYNAMICS<br>-**family dynamics** (8) – influence of matriarch vs. patriarch family set up, separated parents, single mother, young parents, dysfunctional family, lack of family support<br>-**competing crises** (7) – illness in another child, extreme psychosocial dysfunction, drug use by parent, psychiatric disease in parent, lack of time due to work responsibilities<br>-**misguided pressure from extended family** (6) – influence of elder relatives, advice against treatment<br>-**personal character of parents** (6) - inner strength to persevere, lack of emotional commitment, poor motivation, denial<br>-**large family size** (5) – large families, many other children<br>-**gender** (1) - refusal of male to female sibling HSCT<br>-**ranking of the child in the family** (1)<br>-**refusal by patient** (2) – refusal by child or adolescent<br>-**false expectations** (1)<br><br>HEALTH CARE SYSTEM<br>-**health care financing** (14) – lack of governmental support, insurance coverage, treatment costs, loss of social security support, barriers imposed by insurance companies<br>-**access to care in another country** (3) – travel to another country for treatment	VULNERBILITY<br>-**vulnerable populations** (5) – foster child, immigration status, discriminated population<br>-**ethnic background** (4) – indigenous or native patients<br>-**language barriers** (3) – dialects, indigenous languages<br>-**concurrent chronic illness** (2) – mental retardation, underlying illness<br><br>BELIEFS / TRUST<br>-**disbelief in diagnosis** (4) – doubt of diagnosis, denial<br>-**distrust** (4) – of physician or center's capacity<br>-**misbeliefs** (3) – chemotherapy not needed after remission or improved clinical status, cured after resection<br>-**past experience with cancer** (1)<br>-**fear** (1)<br><br>MEDIA<br>-**lack of awareness** (2) – for early detection and for possibilities of treatment in the country<br>-**misinformation by others** (2) – wrong information given by well wishers, counter-counseling by other health workers<br>-**negative impact of media** (2) – advertisement of "new" drugs

[1] Data was retrieved from 194 interpretable responses provided by 104 subjects. Most responses were amenable for grouping into factors. The frequency of each factor is reported in parenthesis to provide its relative frequency.

[2] For comments amenable for grouping into factors, the factor is provided first, followed by its frequency in parenthesis, and some illustrative examples. The list is not inclusive of all comments.
