# Supplementary figures and images for: Determinants of Treatment Abandonment in Childhood Cancer: Results from a Global Survey
Source: PLoS One. 2016 Oct 13;11(10):e0163090. doi: 10.1371/journal.pone.0163090 (PMC5063311; doi:10.1371/journal.pone.0163090)

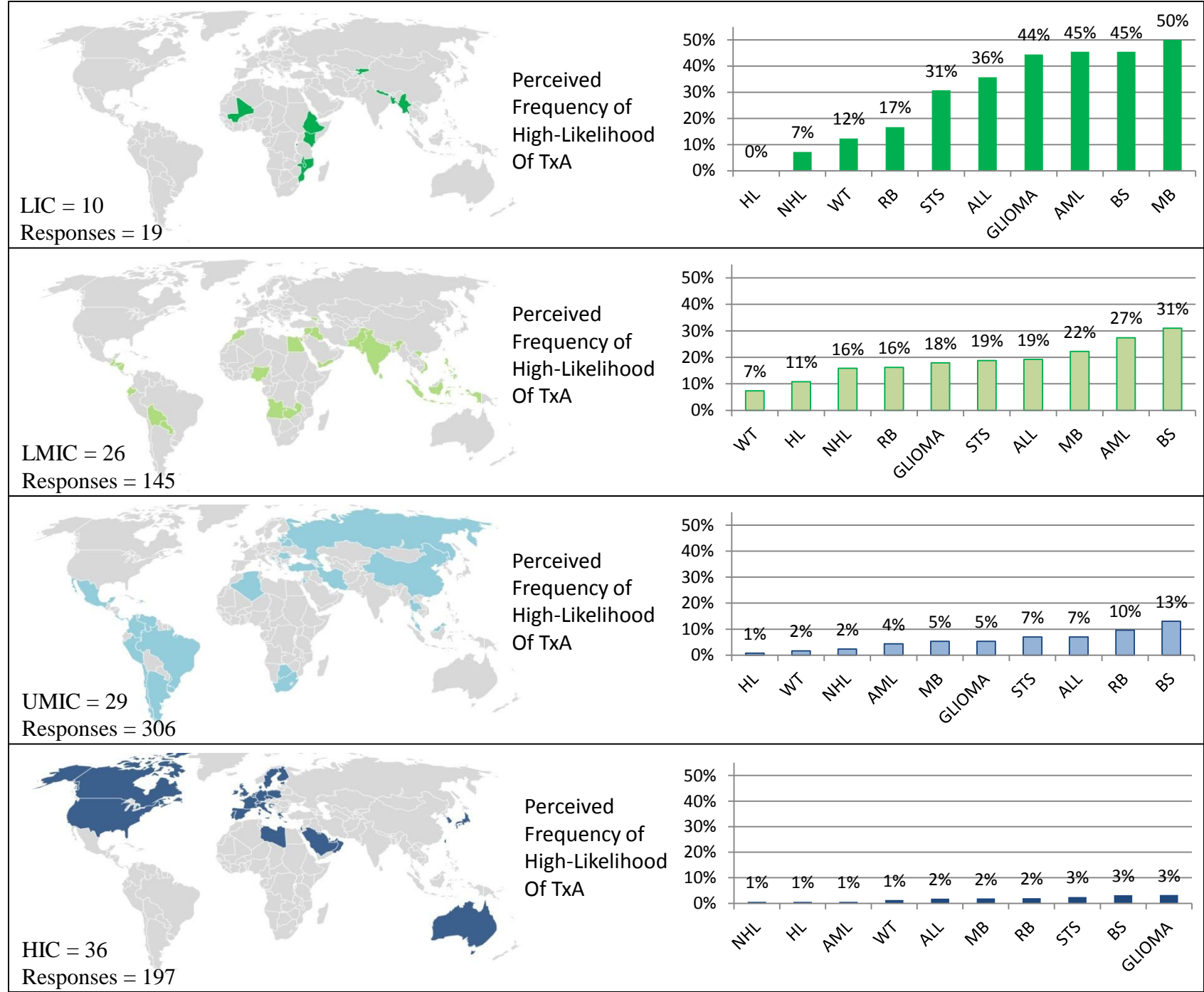

Supplement: S1 Fig — Dark blue, HIC = high-income countries; light blue, UMIC = upper-middle-income countries; light green, LMIC = lower-middle-income countries; dark green, LIC = low-income countries; HL = Hodgkin Lymphoma, NHL = Non-Hodgkin Lymphoma; WT = Wilms tumor; RB = Retinoblastoma; STS = Soft tissue sarcoma; ALL = Acute lymphoblastic leukemia; GLIOMA = Brain glioma; AML = Acute myeloid leukemia; BS = Bone sarcoma; and MB = Medulloblastoma. (PDF) [file pone.0163090.s003.pdf]
