# Supplementary material for: Determinants of Treatment Abandonment in Childhood Cancer: Results from a Global Survey
Source: PLoS One. 2016 Oct 13;11(10):e0163090. doi: 10.1371/journal.pone.0163090 (PMC5063311; doi:10.1371/journal.pone.0163090)
Supplement: S2 Fig — The category “increases likelihood of TxA” entailed report of “increases” or “strongly increases” likelihood of TxA. HIC, high-income countries; LMC, low- and middle-income countries; TxA, treatment abandonment; CAM, complementary and alternative medicine. (PDF) [file pone.0163090.s004.pdf]

	Strongly decreases likelihood of TxA / Decreases likelihood of TxA / No relation to TxA / Increases likelihood of TxA / Strongly increases likelihood of TxA	Increases likelihood (HIC)	Increases likelihood (LMC)	p-value
Low socioeconomic status	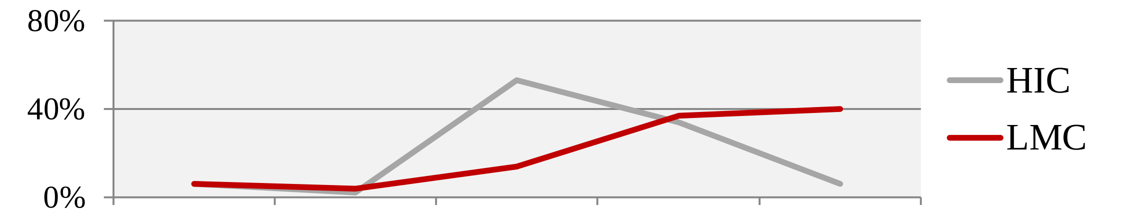	40%	77%	<0.001
Low parental education	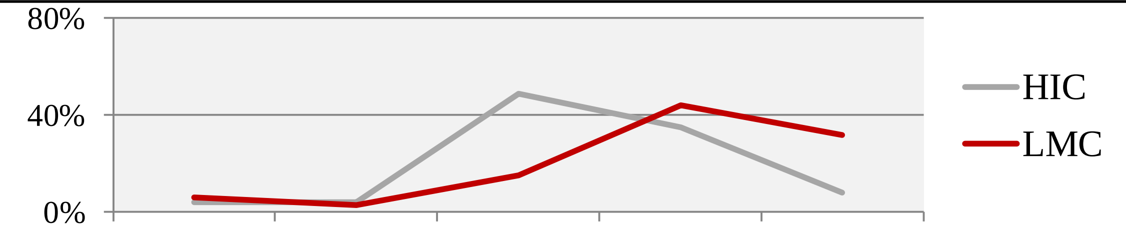	43%	76%	<0.001
Long travel time to center	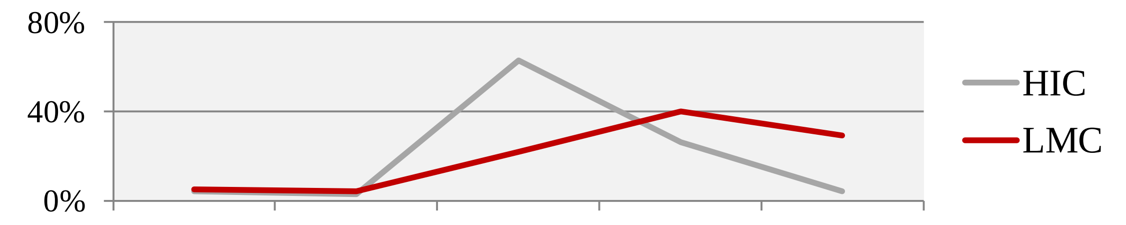	30%	69%	<0.001
Preference for CAM	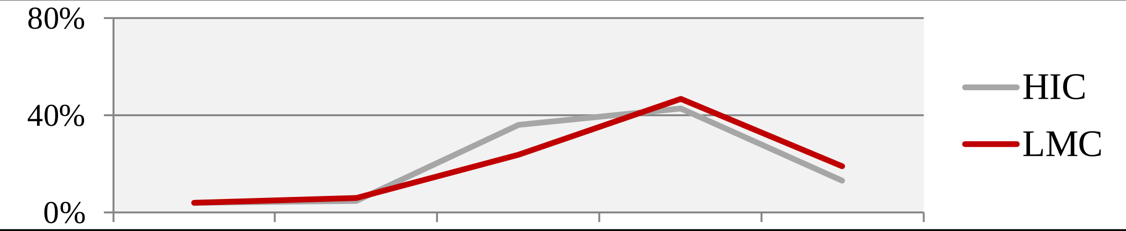	56%	66%	0.03
Adverse effects and toxicity	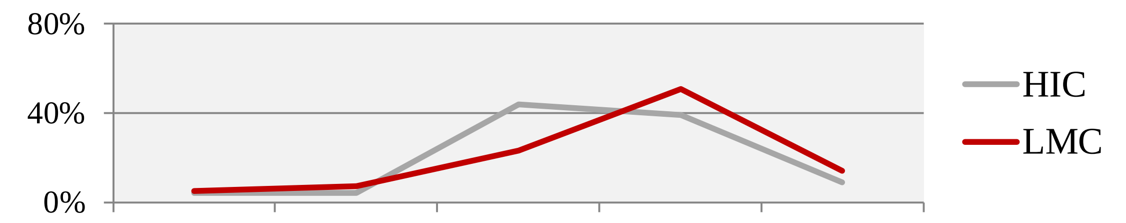	48%	65%	<0.001
Belief in incurability of cancer	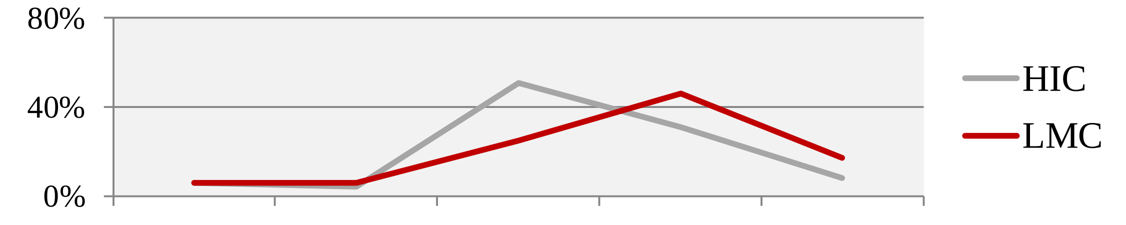	39%	63%	<0.001
Insufficient communication by health professionals	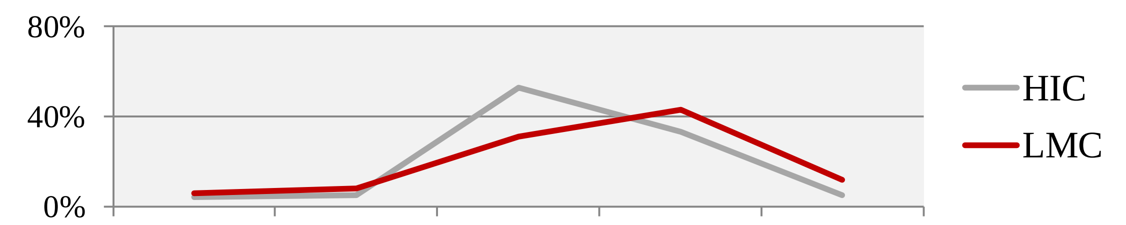	38%	55%	<0.001
Strongly held faith or religious beliefs	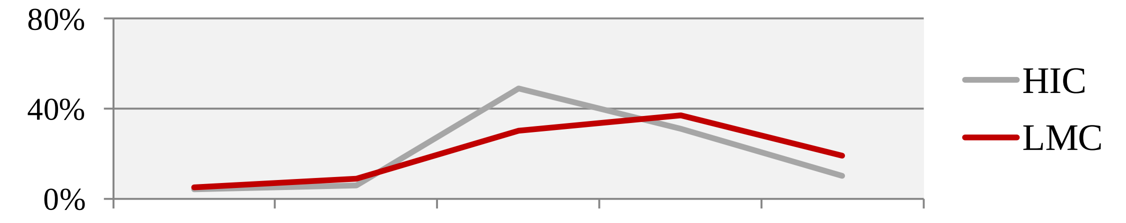	41%	56%	<0.001
Painful diagnostic or therapeutic procedures	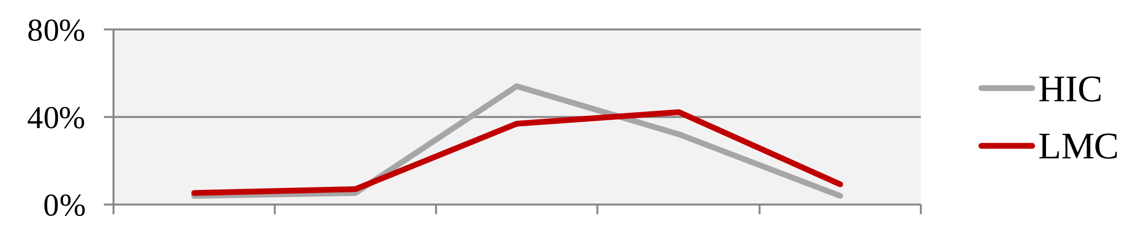	36%	51%	0.001
Older child or adolescent	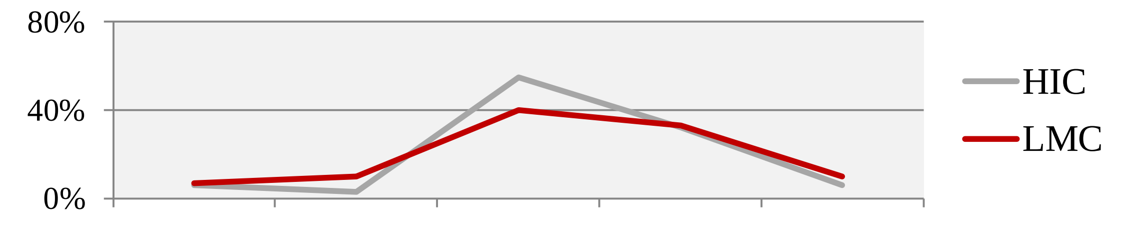	38%	43%	0.2
Undernourished child	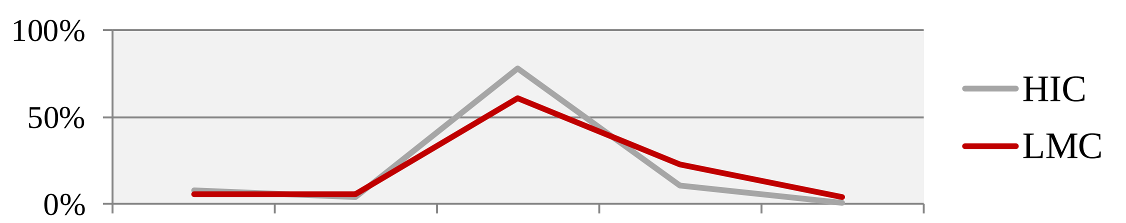	12%	27%	<0.001
HIV positive child	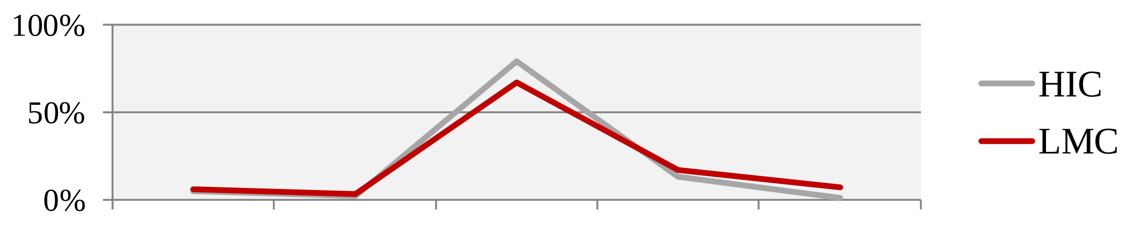	14%	24%	0.009
Younger child	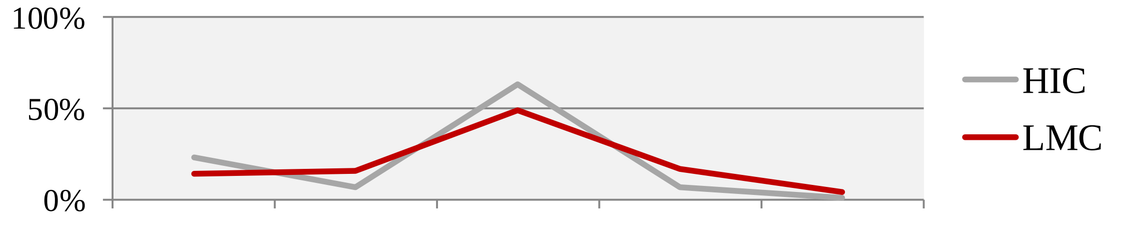	8%	21%	<0.001
Female child	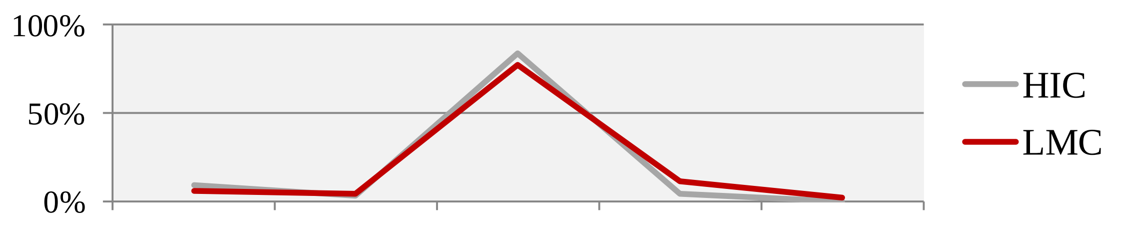	4%	13%	0.001
Male child	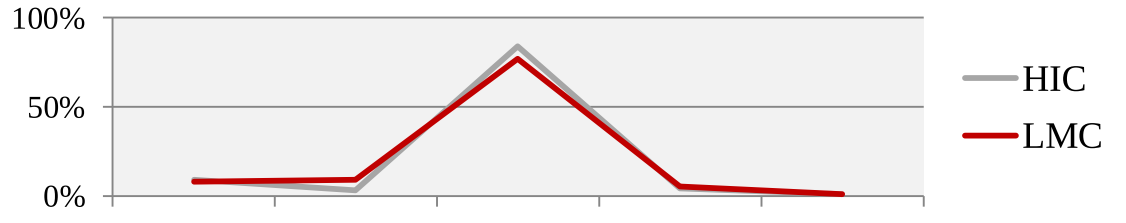	5%	6%	0.3
